# The Incorporation of Copper-Doped Bioactive Glass Nanoparticles into Resin Composites Improves Their Biological, Mechanical and Adhesive Properties

**DOI:** 10.3290/j.jad.c_2014

**Published:** 2025-05-07

**Authors:** Romina Aliaga-Gálvez, Mario Felipe Gutiérrez, Benjamín Valenzuela, Saulo Geraldeli, Gabriel Abuna, Carolina Inostroza, Cristian Bravo, Gabriel Cochinski, Alessandro D. Loguercio

**Affiliations:** a Romina Aliaga-Gálvez PhD student, Universidad de los Andes, Chile, Facultad de Odontología, Santiago, Chile. Conceptualization, data curation, methodology, writing – original draft preparation.; b Mario Felipe Gutiérrez Associate professor, Universidad de los Andes, Chile, Facultad de Odontología, Santiago, Chile; Universidad de los Andes, Chile, Centro de Investigación e Innovación Biomédica (CIIB), Santiago, Chile. Conceptualization, data curation, formal analysis, funding resources, supervision, visualization, writing – original draft preparation, writing – review and editing.; c Benjamín Valenzuela Dentist, Universidad de los Andes, Chile, Facultad de Odontología, Santiago, Chile. Conceptualization, data curation, methodology, writing – original draft preparation.; d Saulo Geraldeli Associate professor, Department of General Dentistry, School of Dental Medicine – East Carolina University, Greenville, NC, USA. Methodology, project administration, writing – review and editing.; e Gabriel Abuna Assistant professor, Department of General Dentistry, School of Dental Medicine – East Carolina University, Greenville, NC, USA. Methodology, project administration, writing – review and editing.; f Carolina Inostroza Associate professor, Universidad de los Andes, Chile, Facultad de Odontología, Santiago, Chile; Universidad de los Andes, Chile, Centro de Investigación e Innovación Biomédica (CIIB), Santiago, Chile. Methodology, project administration, writing – review and editing.; g Cristian Bravo Dean, Universidad de los Andes, Chile, Facultad de Odontología, Santiago, Chile. Methodology, project administration, funding resources, supervision, writing – review and edit.; h Gabriel Cochinski Researcher, Department of Restorative Dentistry, School of Dentistry, State University of Ponta Grossa, Ponta Grossa, Brazil. Conceptualization, data curation, methodology, writing – original draft preparation.; i Alessandro D. Loguercio Associate professor, Department of Restorative Dentistry, School of Dentistry, State University of Ponta Grossa, Ponta Grossa, Brazil. Conceptualization, data curation, formal analysis, funding resources, supervision, visualization, writing – original draft preparation, writing – review and editing.

**Keywords:** copper, bioactive glass, microtensile bond strength and nanoleakage, nanoparticles, resin composite

## Abstract

**Purpose:**

This study aims to develop and characterize copper-doped bioactive glass nanoparticles (BG/CuNp), and to evaluate the effects of their addition into a resin composite on antimicrobial activity (AMA), cytotoxicity (CTX), ultimate tensile strength (UTS), Knoop microhardness (KHN), as well as immediate resin-dentin microtensile bond strength (μTBS), nanoleakage (NL) and in-situ degree of conversion (DC).

**Materials and Methods:**

BG/CuNp were added to a resin composite at different concentrations (0% [control]; 5, 10 and 20 wt%). The AMA was evaluated against *Streptococcus mutans*. For CTX, the Gingival mesenchymal stem cells (GMSC) cell line was used. For UTS and KHN, specimens were tested after 24 h and 28 days. For bonding evaluation, a universal adhesive was applied on flat dentin surfaces, experimental resin composite build-ups were prepared, and specimens were sectioned to obtain resin–dentin sticks. These were evaluated for μTBS, NL and DC after water storage. Data were submitted to statistical analyses (α = 0.05).

**Results:**

The addition of 5% and 10% of BG/CuNp increases AMA (P < 0.05), while the CTX remained unchanged with resin-containing BG/CuNp (P > 0.05). UTS and KHN remained stable with the addition of 5% and 10% of BG/CuNp at 24 h, but showed significantly higher values compared to the control after 28 d (P < 0.05). μTBS and *in-situ* DC remained unchanged with BG/CuNp addition, regardless of the concentration added. However, significantly lower NL was observed for BG/CuNp groups (P < 0.05).

**Conclusion:**

The addition of BG/CuNp in the tested concentrations into a resin composite may be an alternative to provide antimicrobial activity and improve the integrity of the hybrid layer, without compromising biological, adhesives and mechanical properties.

Due to the increasing demand for esthetic dental restorations, resin composites are gaining significant space in restorative dentistry. They are widely used to restore the anatomical shape of teeth that have suffered damage from the removal of decayed tissue or fractures.^58^ Resin composites are primarily composed of a polymeric matrix of photo-initiators and monomers, reinforcing fillers, and a silane coupling agent.^18,37^ This composition results in a restorative material that offers several key advantages, such as use for direct filling, convenient handling, photo-polymerization capabilities, and minimally invasive preparation techniques.^3,9,12^ As a result, resin composites have become the preferred choice for clinicians when performing dental restorations.^11,34,56^

However, concerns persist regarding the limited durability and clinical success of contemporary resin composite restorations.^23,47^ A literature review of clinical studies has reported annual failure rates up to 6.3%,^10^ with cumulative failure rates reaching 58% after 10 years.^64^ A systematic review of the literature has suggested that the average lifespan of posterior resin composite restorations is around 6 years.^15^ Furthermore, it is reported that 50–70% of newly placed restorations are replacements for failed pre-existing ones, resulting in significant dental care expenses related to the replacement of these restorations. The main causes for the failure of resin composite restorations include secondary caries (20–44%) and restoration fracture,^16,38^ which negatively affect their clinical success3. Unfortunately, these issues have persisted for over 30 years.^19^

On the other hand, the subsequent replacement or repair of a restoration to address secondary caries results in the loss and weakening of tooth structure, leading to an increase in cavity volume and requiring more complex restoration. This process contributes to the so-called restorative death spiral, which may ultimately result in tooth loss.^14,17,25^ Consequently, the reduced longevity and the need to replace these restorations with more complex ones account for over 5 billion dollars in annual expenses in the United States of America. In Latin America, where the caries risk is higher compared to the United States of America,^46^ it is expected that the situation may be equally problematic, if not worse, with the increased demand for health services being posing a significant public policy challenge.

These factors underscore the importance of developing materials with both antibacterial and remineralizing properties to enhance the durability of the resin-tooth interface, without compromising the mechanical properties of the resin composite material.^8,26^ Copper nanoparticles (CuNp) have demonstrated effectiveness against both Gram-positive and Gram-negative bacteria.^55^ Beyond their significant antimicrobial activity, CuNps are cost-effective, making their synthesis a favorable option for enhancing material performance at a better cost-benefit ratio.

On the other hand, bioactive glasses represent promising additions to restorative dentistry due to their ability to elevate local pH, release beneficial ions, such as Ca^2+^, PO4^3−^and F^−^, and promote the formation of apatite in the resin-tooth interface, which aids in remineralization. Although these elements have been incorporated individually into dental materials, and their combination has been successfully tested in scaffolds for bone regeneration,^33,62^ there is no existing research on the incorporation of both elements together in a resin composite system. The concept behind this approach is to harness the antimicrobial and remineralizing properties of bioactive glasses to develop a resin composite system that exhibits all these beneficial characteristics. No incompatibilities between these components have been reported in the literature,^33,62^ suggesting that combining them could enhance the stability and durability of the resin-tooth interface, thereby improving resistance to secondary caries without compromising the mechanical properties of the polymeric material. With this in mind, we designed this *in vitro* study to evaluate the effects of incorporating various concentrations of copper-doped bioactive glass nanoparticles (BG/CuNp) into a commercial resin composite system. This primary study focuses on assessing antimicrobial activity (AMA), cytotoxicity (CTX), ultimate tensile strength (UTS), Knoop microhardness (KHN), as well as immediate resin-dentin microtensile bond strength (μTBS), nanoleakage (NL) and *in-situ* degree of conversion (DC). Thus, the following null hypotheses were tested: 1st) there is no significant difference in the biological properties evaluated (AMA and CTX) when a resin composite system with different BG/CuNp concentrations is used (0%, 5%, 10%, and 20%); 2nd) there is no significant difference in the mechanical properties evaluated (UTS and KHN) when a resin composite system with different BG/CuNp concentrations is used; and 3rd) there is no significant difference in the adhesive properties evaluated (μTBS, NL and DC) when a resin composite system with different BG/CuNp concentrations is used.

## MATERIALS AND METHODS

### Copper-Doped Bioactive Glass Nanoparticles: Synthesis and Characterization

The synthesis of a four-component Na_2_O–CaO–P_2_O_5_–SiO_2_ bioglass powder was accomplished using a modified Stӧber process 60. The reagents used in the synthesis included calcium nitrate tetrahydrate (Ca (NO_3_)₂·4H_2_O), sodium nitrate (NaNO_3_), triethyl phosphate ((C2H5)3PO4), copper chloride (CuCl), and tetraethyl orthosilicate (Si(OC_2_H_5_)_4_, reagent grade, 98%, TEOS, Sigma Aldrich, Darmstadt, Germany). Ammonium hydroxide (ACS Reagent, 28.0–30.0% NH_3_ basis) served as the catalyst. The synthesis began by preparing a solution containing 200 mL of deionized water and 1M ammonium hydroxide in a round-bottom vessel. TEOS was added to this solution, forming the initial reaction mixture. The subsequent reagents were introduced to the TEOS solution in the following order: (C_2_H_5_)_3_PO_4_, NaNO_3_, and Ca(NO_3_)_2_·_4_H_2_O. The reaction mixture was maintained at 60°C under constant stirring with a magnetic bar for 3 h. Once the reaction was complete, the bioactive glass precipitate was collected by centrifugation. The precipitate was washed three times with ethanol and distilled (DI) water at 3500 rpm for 2 min per cycle. After removing the supernatant, the solid pellet was dried in a vacuum oven at 60°C for 24 h. To obtain the bioactive glass nanoparticles, the dried samples underwent annealing at 680°C for 3 h. These thermal treatment parameters were selected to optimally remove nitrates and incorporate calcium ions into the silica glassy network without causing full crystallization. The synthesis of Cu-modified bioglass employing partial calcium replacement was done by adding copper chloride (CuCl) in the 2.5 mol% concentration range to the TEOS solution.

The copper-doped bioactive glass nanoparticles were characterized by scanning electron microscope (SEM) and dynamic light scattering (DLS) for size measurement range, and energy dispersive X-ray (EDX) analysis.

### Formulation of the Experimental Resin Composites

We formulated experimental resin composites using a commercial resin composite system (Opallis; FGM Dental Group, Joinville, SC, Brazil). Four experimental resin composites were formulated according to the addition of different concentrations of BG/CuNp (wt%): 0% (control, commercial material); 5%, 10%, and 20%. These concentrations were used based on results from a pilot study (data not shown). The incorporation into the resin composite was done in a dark room.^21^

### Antimicrobial Activity

#### Bacterial strain and growth conditions

The experiments were performed using *Streptococcus mutans* (reference strain ATCC 23175) obtained from a repository. The bacterial culture was stored at −80ºC in BHI medium (Difco Laboratories, Detroit, MI, USA) containing 20% (v/v) glycerol until needed.

#### Specimen preparation

After isolating a metallic matrix (10.0 mm diameter, 0.5 mm thick) with petroleum jelly, we dispensed the resin composite until filling the mold. Under a plastic matrix strip, the adhesive was light-cured for 40 s with an LED light source at 1000 mW/cm^2^ (VALO, Ultradent Products, South Jordan, UT, USA), in close contact with each disc. After polymerization, the specimens were removed from the mold and polished with 1000–, 1500–, 2000– and 3000-grit SiC paper to remove the oxygen-inhibition layer. After storage in a dark vial for 24 h, all specimens were sterilized under UV light for 10 min per side in a Clean Bench (ZHJH-C1106C; Zhicheng, Shanghai, China). The discs were stored in 24-well plates with 1 mL of sterile distilled water until the experiment. Five discs per group were tested.

#### Antibacterial activity of resin composites against S. mutans biofilm

Overnight cultures of *S. mutans* were grown in BHI supplemented with 20% (w/v) sucrose at 37ºC and 5% CO_2_, measured regarding optical density (OD600 ≥ 0.800) and used as biofilm-grown inoculum. The discs were horizontally placed in 24-well plates and inoculated with an *S. mutans* culture of approximately 2 × 106 CFU/mL (colony-forming units per mL). Additionally, one sterile disc was incubated with a sterile medium and used as a control. After 24 h, the disks were washed two times in 1× PBS (Cytiva, Marlborough, MA, USA) at 200 rpm in a shaker at 37ºC (Thermoline, Wetherill Park, Australia) to remove non-adhering bacteria.

The biofilm was removed from the discs by vibration (Vortex mixer; MRC, Holon, Israel). The biofilm was then serially diluted in PBS in Eppendorf tubes and plated on BHI agar to count the viable cells (CFU/mL) and, consequently, evaluate the antibacterial effectiveness of the tested resin composites. The count of the viable cells (CFU/mL) was measured in all pictures using the public domain Image J software, a Java-based image processing software package developed at the National Institutes of Health (NIH).^54^

### Cytotoxicity Evaluation Using GMSCs Cell Line

#### Cell culture

Gingival mesenchymal stem cells (GMSCs) previously obtained as described in a previous article^43^ were used to assess the cytotoxicity of the resin composite groups. Discs of 10 mm diameter and 1.5 mm wide for each group were developed for this test. Cells were cultured in Dulbecco’s Modified Eagle’s Medium (DMEM; Thermo Fisher Scientific, Dreieich, Germany) supplemented with 10% Fetal Bovine Serum (FBS; BI Biological Industries) and 1% penicillin-streptomycin solution (Thermo Fisher Scientific, Dreieich, Germany), 50 µg/mL Plasmocin® (InvivoGen, San Diego, CA, USA), 5 µg/mL Fungizone® (Bristol-Myers Squibb, Princeton, NJ, USA) in a humidified atmosphere containing 5% CO_2_ at 37°C in an incubator (Sanyo, Incubator MCO-18M, Kyoto, Japan).

#### Assessment of cell proliferation using the WST-1^®^ assay

Cell proliferation was evaluated using the WST-1® Cell Proliferation Reagent (Roche Diagnostics, Germany). GMSCs (10^4^ cells per well) were cultured over time under standard conditions in 24-well plates. Discs of each experimental resin composite (as previously described) were sterilized under UV light for 10 min per side in a Clean Bench (ZHJH-C1106C; Zhicheng, Shanghai, China) before co-culturing with GMSCs and 100 μl cell culture medium. Incubation with plain culture medium was used as a 100% viability control, while chlorhexidine 2% (Difem Pharma, Santiago, Chile) was used as a positive control.

After 24 h, 7 days and 14 days of incubation, the culture medium was removed and transferred to 96-well plates, where 10 µL of WST-1® reagent was added. The plates were then incubated at 37ºC for 2 h. Absorbance was measured in triplicate at 450 nm, with a MultiSkan Sky microplate reader (Thermo Fisher Scientific, Dreieich, Germany).

#### Ultimate tensile strength

A metallic matrix with an hourglass shape (10 mm long, 2 mm wide, 1 mm deep and a cross-sectional middle area of approximately 1.5 mm^2^) was used to construct the specimens. After isolating the metallic matrix with a very thin layer of petroleum jelly, we applied the resin composite until it filled the mold. Under a plastic matrix strip, the resin composite specimens were light-cured for 40 s with a LED light source at 1000 mW/cm^2^ (VALO, Ultradent Products, South Jordan, UT, USA), in close contact with each hourglass-like specimen. After polymerization, the specimens were removed from the mold and polished with 600-grit SiC paper to remove the adhesive excesses and the oxygen-inhibition layer.

Half of the samples were tested 24 h after preparation, and the other half were tested 28 days after water storage at 37ºC. The cross-sectional area of each specimen was measured with a digital caliper to the nearest 0.01 mm and recorded for subsequent calculation of the ultimate tensile strength values (Absolute Digimatic, Mitutoyo, Tokyo, Japan). Each specimen was attached to a modified device with cyanoacrylate resin (IC-Gel, bSi, Atascadero, CA, USA) and subjected to a tensile force at 0.5 mm/min. Five specimens were tested per group and for evaluation time.

#### Knoop microhardness

Five resin discs (10 mm diameter and 1.5 mm wide) of each group and evaluation time were produced as described for the ultimate tensile strength. After preparation, the specimens were stored in a dark vial for 24 h and 28 days before microhardness measurement. The specimens were then placed in an HMV-2 microhardness tester (Shimadzu; Tokyo, Japan) equipped with a Knoop indenter. Five measurements were performed on each specimen with a 50-g load for 15 s.^7^ The first measurement was taken at the center of the resin composite disc. The other four measurements were performed 100 µm and 200 µm to the left and right of the first one. The values obtained for the same specimen were averaged for statistical purposes.

#### Teeth preparation and bonding procedures

Sixty-four caries-free extracted human third molars, collected from patients aged 18 to 35 years, were used. The teeth were collected after obtaining the patient’s informed consent. The local research committee and the scientific ethics committee reviewed and approved this study under protocol number CEC 2022007. Teeth were disinfected in 0.5% chloramine, stored in distilled water, and used within 3 months after extraction. A flat dentin surface was exposed on each tooth after wet grinding the occlusal and surrounding enamel with 180-grit SiC paper. The enamel-free, exposed dentin surfaces were further polished with 600-grit silicon-carbide paper for 60 s to standardize the smear layer.

A universal adhesive was applied in either etch and rinse (ER) or self-etch (SE) mode, according to the manufacturer’s instructions (Table 1). In ER mode, the dentin surface was acid-etched with 37% phosphoric acid for 15 s (Condac, FGM Dental Group, Joinville, SC, Brazil), rinsed with water for 15 s, and dried with absorbent paper, leaving the dentin surface slightly wet. After the bonding procedures, resin composite blocks (Opallis, FGM Dental Group, Joinville, SC, Brazil) were built up on the bonded surfaces in three increments, each 1.0 mm thick, with each increment individually light activated for 20 s with a LED light source at 1000 mW/cm^2^ (VALO, Ultradent Products, South Jordan, UT, USA). A single operator carried out all bonding procedures in an environment with controlled temperature and humidity. Eight teeth were used for each experimental group.

**Table 1:** Composition, batch number, and application mode of universal adhesive system and resin composites in the different groups

Universal adhesive system (batch number) and pH	Composition (*)	Etch-and-rinse mode	Self-etch mode
Condac 37 (CON – FGM Dental Group, Joinville, Santa Catarina, Brazil) (010823)	37 % phosphoric acid	1. Apply phosphoric acid for 15 s. 2. Wash the surface with plenty of water. 3. Dry the cavity so that the dentin does not get dehydrated, but without the accumulation of water on the surface.	
Ambar Universal APS (AMU – FGM Dental Group, Joinville, Santa Catarina, Brazil) (070722) pH= 2.6–3.0	10-MDP, methacrylic monomers, photo-initiator APS, CQ, silica nanoparticles, ethanol, co-initiators, and stabilizers	1. Apply a first layer vigorously rubbing the adhesive with the micro applicator for 10 s. 2. Next, apply a second layer of adhesive for 10 s, spreading the product. 3. Evaporate excess solvent by thoroughly air-drying with an air syringe for 10 s. 4. Light cure for 10 s at 1000 mW/cm^2^.	1. Apply a first layer vigorously rubbing the adhesive with the micro applicator for 10 s. 2. Next, apply a second layer of adhesive for 10 s, spreading the product. 3. Evaporate excess solvent by thoroughly air-drying with an air syringe for 10 s. 4. Light cure for 10 s at 1000 mW/cm^2^.
Opallis (OPA – FGM Dental Group, Joinville, Santa Catarina, Brazil) (240822)	Bis-GMA monomers, Bis-EMA, TEGDMA, UDMA, CQ, co-initiator, silanized barium-aluminum silicate glass (particle size of 0.5 μm, 79.8 wt%), pigments and silica	1. Placed in increments of 1 mm (three layers). 2. Light cure for 20 s each layer at 1000 mW/cm^2^.	1. Placed in increments of 1 mm (three layers). 2. Light cure for 20 s each layer at 1000 mW/cm^2^.
(*) 10-MDP = methacryloyloxydecyl dihydrogen phosphate; CQ: canforquinone; Bis-GMA = bisphenol A diglycidylmethacrylate; Bis-EMA: ethoxylated bisphenol A diglycidylmethacrylate; TEGDMA = triethyleneglycol dimethacrylate; UDMA = urethane dimethacrylate.

After storing the bonded teeth in distilled water at 37ºC for 24 h, they were longitudinally sectioned in both the ‘x’ and ‘y’ directions across the bonded interface using a diamond saw in a cutting machine (Mecatome T205; Presi, France), under water cooling at 300 rpm, to obtain resin–dentin sticks with a cross-sectional area of approximately 0.8 mm^2^. The number of premature failures (PF) per tooth during specimen preparation was recorded. The cross-sectional area of each specimen was measured with a digital caliper to the nearest 0.01 mm and recorded for subsequent calculation of the microtensile bond strength values (Absolute Digimatic, Mitutoyo, Tokyo, Japan). The resin–dentin bonded sticks from each tooth were then divided as follows:

Two sticks were used for nanoleakage evaluation.
Two sticks were used to measure the immediate in-situ degree of conversion.
The remaining sticks were subjected to the microtensile bond strength test.

#### Microtensile bond strength testing

Each stick was attached to a modified device for microtensile bond strength test using cyanoacrylate resin (IC-Gel, bSi Inc., Atascadero, CA, USA) and subjected to a tensile force in a universal testing machine (OM150; Odeme Dental Research, Luzerna, SC, Brazil) at a rate of 0.5 mm/min. The failure mode was evaluated under an optical microscope (SZH-131, Olympus; Tokyo, Japan) at 40× magnification and classified as cohesive in dentin (failure exclusively within cohesive dentin – CD); cohesive in resin (failure exclusively within cohesive resin – CR); adhesive (failure at resin/dentin interface – A), or mixed (failure at resin/dentin interface, including cohesive failure of the neighboring substrates, M). The number of PF was recorded, and was not included in the average mean bond strength.

#### Nanoleakage evaluation

Before performing the nanoleakage test, a pilot test was conducted to evaluate whether the presence of bioactive glass and copper in the resin composite could impair the visualization of silver nitrate uptake. For this purpose, SEM images of resin–dentin interfaces from all groups were taken without immersion in silver nitrate. Even in adhesive interfaces with the highest concentrations, bioactive glass and copper were not observed using the same parameters described above. Therefore, the results of nanoleakage test reflect the amount of silver uptake into unpolymerized areas and/or nanospaces not infiltrated by the resin adhesive, but not the presence of bioactive glass or copper in the hybrid layer.

After this preliminary test, all resin–dentin sticks selected for this test were coated with two layers of nail varnish, applied up to within 1 mm of the bonded interfaces. The resin–dentin sticks were immersed in a 50 wt% ammoniacal silver nitrate solution in total darkness for 24 h, rinsed thoroughly in distilled water, and immersed in photo-developing solution for 8 h under fluorescent light to reduce silver ions to metallic silver grains within voids along the bonded interface.

Specimens were mounted on aluminum stubs, polished with 1000-, 1500-, 2000- and 3000-grit SiC paper, and 1 and 0.25 µm diamond paste (Buehler Ltd., Lake Bluff, IL, USA). Then, they were ultrasonically cleaned, air-dried and gold sputter-coated (MED 010, Balzers Union, Balzers, Liechtenstein). The interfaces were observed in a SEM in backscattered mode at 15 kV (VEGA 3 TESCAN, Shimadzu, Tokyo, Japan).

To standardize image acquisition, three pictures were taken of each specimen. The first picture was taken in the center of the resin–dentin stick, while the other two pictures were taken 0.3 mm to the left and right of the first one. As two resin–dentin sticks per tooth were evaluated and a total of eight teeth were used for each experimental condition, a total of 48 images were evaluated per group. A technician, who was blinded to the experimental conditions, took all the images. The relative percentage of nanoleakage within the adhesive and hybrid layer areas was measured in all pictures using the public domain Image J software, a Java-based image processing software package developed at the NIH.^54^

#### In-situ degree of conversion (DC) within resin layers

All resin–dentin sticks selected for this test were wet polished using 1000, 1500; 2000; 2500 and 3000-grit SiC paper for 30 s each. The specimens were ultrasonically cleaned for 10 min and then placed into the micro-Raman equipment. The DC measurements were performed using a micro-Raman spectrometer (Horiba Scientific, Tokyo, Nagoya, Japan).

The micro-Raman spectrometer was first calibrated for zero and then for coefficient values using a silicon specimen. Specimens were analyzed using the following micro-Raman parameters: 20-mW Neon laser with 532-nm wavelength, spatial resolution of ≈ 3 µm, spectral resolution ≈ 5 cm^-1^, accumulation time of 30 s with 6 co-additions, and magnification of 100× (Olympus UK, London, UK) to achieve a beam diameter of ≈ 1 µm. Spectra were taken at the resin–dentin interface, in the middle of the hybrid layer within the intertubular dentin, at three different sites for each specimen, and the values were averaged for statistical purposes. Spectra of uncured adhesives were taken as the reference.

The ratio of the double-bond content of monomer to polymer in the resin was calculated according to the following formula: DC (%) = (1 – R_cured_/R_uncured_) × 100, where R is the ratio of aliphatic and aromatic peak areas at 1639 cm^–1^ and 1609 cm^–1^ in cured and uncured adhesives.

#### Statistical analysis

The data were first analyzed using the Kolmogorov–Smirnov test to assess whether the data followed a normal distribution, as well as Barlett’s test for equality of variances to determine if the assumption of equal variances was valid. Data from the antibacterial activity test did not present a normal distribution. Therefore, CFU values were transformed into a log and analyzed by the non-parametric independent samples Kruskal–Wallis test, accompanied by Dunn’s multiple comparisons test. On the other hand, after confirming the normality of the data distribution and the equality of the variances, data for UTS and microhardness (KNH) were subjected to a two-way ANOVA (resin composite vs time). The μTBS (MPa), nanoleakage (%), and *in-situ* degree of conversion (%) data were subjected to two-way ANOVA (resin composite vs adhesive strategy). Tukey’s post hoc test was used for pair-wise comparisons (α = 0.05) using the Statistica for Windows software (StatSoft, Tulsa, OK, USA).

## RESULTS

### Characterization of Copper-Doped Bioactive Glass Nanoparticles

**Fig 1:**
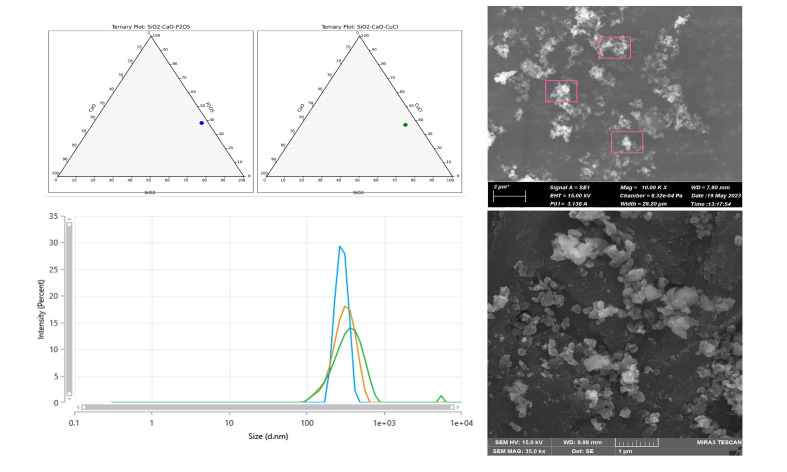
Scanning electron microscopy (SEM) and dynamic light scattering (DLS) images of copper-doped bioactive glass nanoparticles, demonstrating their nanometer size.

**Fig 2:**
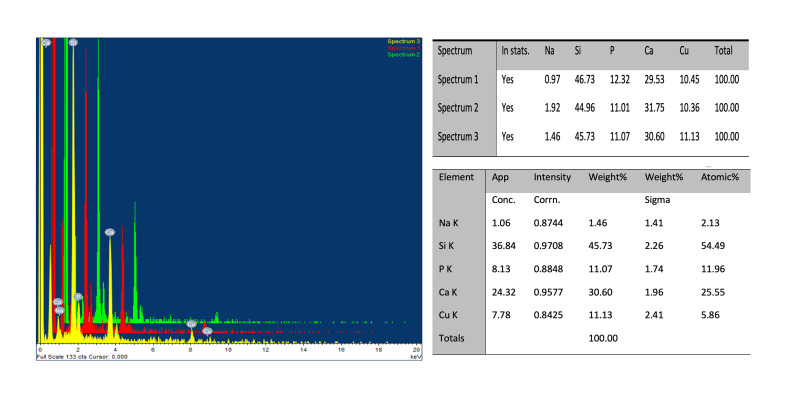
EDX spectrum from a selected area of copper-doped bioactive glass nanoparticle powder. The table summarizes the elemental composition of the sample area outlined in the figure.

The SEM and DLS (Fig 1) confirm that the copper-doped bioactive glass nanoparticles are within the nanometer size range. ZetaSizer measurements indicate a mean size of 321 ± 39 nm, which may be attributed to the agglomeration of the copper-doped bioactive glass nanoparticles in deionized water. As showed in the EDX spectrum of a representative specimen (Fig 2), the samples contain a high percentage of copper, calcium, phosphate, and silica atoms, without contamination from other elements.

### Antimicrobial Activity

The results of antimicrobial activity against *S. mutans* for the different concentrations of BG/CuNp incorporated into a resin composite are presented in Table 2. Only the 5% and 10% groups showed significantly higher antibacterial properties against *S. mutans* than the control (P < 0.05).

**Table 2:** Median and interquartile range of antimicrobial activity (CFU/mL), and means and standard deviations of ultimate tensile strength (MPa) and Knoop microhardness (KHN) obtained at 24 h and 28 days in each experimental condition (*)

BG/CuNp concentration (%)	Antimicrobial activity (*)	Ultimate tensile strength (+)	Microhardness (+)
24 h	28 days	24 h	28 days
**0 (control)**	3.2 × 105 (2.6 × 105 – 3.4 × 105)^B^	17.94 ± 4.31 **AB**	10.45 ± 0.84 **D**	74.24 ± 0.84 **b**	69.52 ± 1.32 **c**
**5%**	1.4 × 105 (8.0 × 104 – 1.9 × 05)^A^	18.95 ± 1.33 **A**	15.18 ± 1.42 **ABC**	76.21 ± 1.70 **ab**	74.26 ± 0.44 **b**
**10%**	9.5 × 104 (5.0 × 104 – 3.3 × 105)^A^	18.25 ± 1.85 **A**	16.90 ± 2.15 **AB**	78.57 ± 1.92 **a**	75.47 ± 1.92 **ab**
**20%**	2.7 × 105 (4.1 × 104 – 6.4 × 105)^AB^	13.52 ± 1.59 **BCD**	12.29 ± 2.26 **CD**	69.28 ± 0.68 **c**	66.88 ± 0.88 **c**
(*) Comparisons are valid only within test. Means identified with the same capital subscripted letters are statistically similar (Kruskal–Wallis; Dunn’s multiple comparisons test, P ≥ 0.05). (+) Comparisons are valid only within test. Means identified with the same capital or lowercase letters are statistically similar (two-way ANOVA; Tukey’s test, P ≥ 0.05).

### *In-vitro* Cytotoxicity

**Fig 3:**
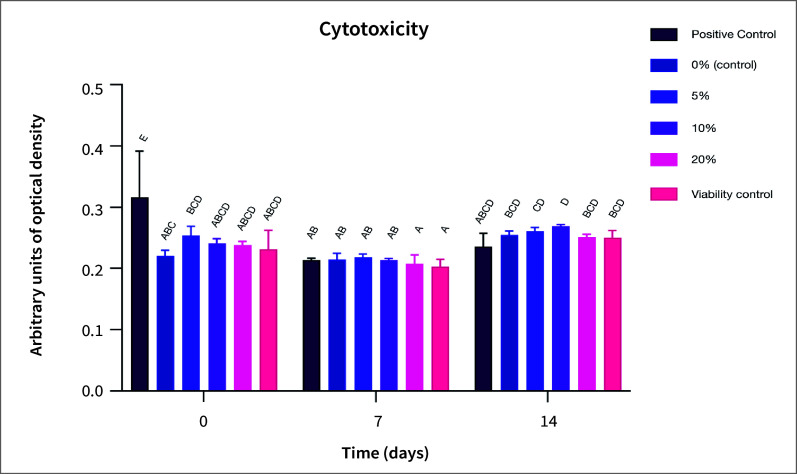
Cytotoxicity of resin-composite-containing copper-doped bioactive glass nanoparticles in GMSCs over time (Day 0, 7 and 14). Data are presented as mean ± SD, with means sharing the same letter indicating no statistically significant difference (Tukey’s test, P ≥ 0.05).

The results of *in-vitro* cytotoxicity against GMSCs for the different concentrations of BG/CuNp, incorporated into a resin composite are shown in Figure 3. At baseline, no differences were observed among experimental groups and the viability control (P > 0.05). However, all experimental groups exhibited low cytotoxicity compared to the positive control (P < 0.01). At day 7 and 14, no differences were observed among the experimental groups (P > 0.05). Additionally, no significant differences were found among experimental groups and the viability control (P > 0.05), nor between the experimental groups and the positive control (P > 0.05).

### Ultimate Tensile Strength

When BG/CuNp were incorporated into the resin composite system, no significant differences between the different groups and the control were detected at baseline (Table 2; P > 0.05). However, the 5% and 10% groups exhibited higher values compared to the 20% group (Table 2; P < 0.01). After 28 days, the 5% and 10% groups showed higher values compared to the control group.

### Knoop Microhardness

When BG/CuNp were incorporated into the resin composite system, the 10% group showed higher values compared to the control group at baseline (Table 2; P = 0.018). In contrast, the 20% group exhibited significantly lower values compared to the control group (Table 2; P = 0.006). After 28 days, the 5% and 10% groups showed significantly higher values compared to both the control group and the 20% group (Table 2; P < 0.05).

### Microtensile Bond Strength Testing

Most of the failures were adhesive/mixed, with only a few cohesive fractures of dentine or resin composite (Table 3). For the etch-and-rinse mode, no significant differences in the microtensile bond strength were observed among all groups (Table 4; P > 0.05). For the SE mode, no significant differences were detected among all groups (Table 4; P > 0.05).

**Table 3:** Fracture pattern, pre-tested failures, and number of resin–dentin beams (n) tested in the microtensile bond strength test in each experimental condition (*)

ZnO/Cu concentration (%)	Etch and rinse	Self etch
Premature failure	n	Fracture pattern	Premature failure	n	Fracture pattern
CD	CR	A	M	CD	CR	A	M
**0 (control)**	2	40	0	0	38	0	1	40	0	0	39	0
**5%**	1	40	0	0	39	0	1	40	0	0	39	0
**10%**	1	40	0	1	38	0	0	40	1	0	38	1
**20%**	2	40	0	2	36	0	3	40	0	1	36	0
(*) Classification of fracture pattern: CD – cohesive dentin; CR – cohesive in resin; A – adhesive; and M – mixed.

**Table 4:** Means and standard deviations of the 24 h microtensile bond strength (MPa), nanoleakage (%) and *in-situ* degree of conversion (%) obtained in each experimental condition (*)

BG/CuNp concentration (%)	Microtensile bond strength	Nanoleakage	*In-situ* degree of conversion
Etch and rinse	Self etch	Etch and rinse	Self etch	Etch and rinse	Self etch
0 (control)	37.2 ± 6.4^A^	36.9 ± 4.5^A^	12.81 ± 3.09 **B**	13.51 ± 2.77 **B**	60.40 ± 1.01 **bc**	61.39 ± 0.81 **abc**
5%	42.4 ± 4.2^A^	41.7 ± 3.5^A^	6.52 ± 0.95 **A**	6.61 ± 2.43 **A**	63.23 ± 1.07 **ab**	64.00 ± 1.94 **a**
10%	42.3 ± 5.4^A^	43.1 ± 2.1^A^	5.15 ± 1.23 **A**	5.49 ± 1.60 **A**	63.83 ± 0.65 **ab**	64.15 ± 1.39 **a**
20%	38.2 ± 6.6^A^	35.0 ± 1.3^A^	5.37 ± 0.98 **A**	5.23 ± 1.47 **A**	58.27 ± 1.37 **c**	58.21 ± 1.41 **c**
(*) Comparisons are valid only within test. Means identified with the same capital or lowercase letter, subscript or not subscripted letters are statistically similar (Tukey’s test, P ≥ 0.05).

### Nanoleakage Evaluation

**Fig 4:**
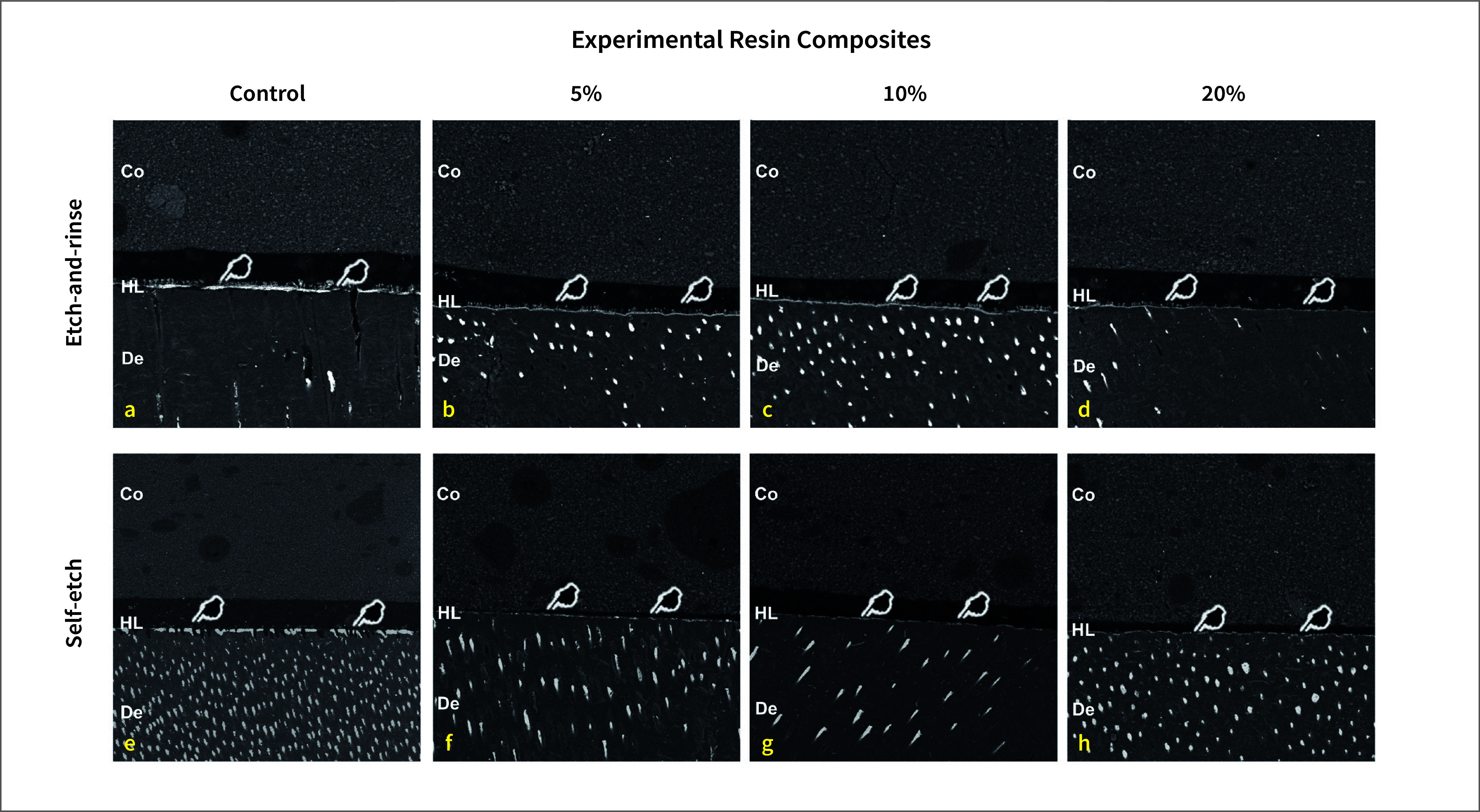
Representative back-scattering SEM images of the resin–dentin interfaces bonded immediately after application under different experimental conditions. (Co = composite; HL = hybrid layer and De = dentin).

For both etch-and-rinse and SE modes, the addition of BG/CuNp resulted in significantly lower nanoleakage values compared to the control group (Fig 4 and Table 4; P < 0.01).

### *In-Situ* Degree of Conversion Within Adhesive/Hybrid Layers

For both etch-and-rinse and SE modes, no significant differences in the *in-situ *degree of conversion were observed among the experimental groups (Table 4; P > 0.05).

## DISCUSSION

The present study was capable to demonstrate that the addition of copper-doped bioactive glass nanoparticles to resin composite systems in concentrations up to 10% could to impact antimicrobial properties and enhance the stability of the resin–dentin interface, without causing significant biological hazards.

The idea behind the incorporation of copper-doped bioactive glass nanoparticles in the formulation of a resin composite system is provide antimicrobial properties against *S. mutans*. In this sense, bacterial species involved in primary caries are also involved in secondary caries,^59^ however the proportion of *S. mutans*, one of the most cariogenic bacteria associated with primary and secondary caries is significantly higher in the biofilm from dentin and enamel restored with resin composite than sound and non-restored tooth.^5,59^ Furthermore, resin composite may cause shifts in the dental biofilm, promoting the growth of more cariogenic bacteria. This occurs because unreacted monomers released from partially polymerized resin composite restorations can enhance the growth and cariogenic potential of the biofilm that forms on these restorations.^29,30,51^ Additionally, resin composites are unable to increase the local pH, which helps the growth of more acidogenic and aciduric bacteria, increasing the cariogenicity of the biofilm. Combined with their lack of antimicrobial properties, this inability to buffer acids may explain why resin composites are more prone to secondary caries.^39^

In this sense, several studies have shown the antimicrobial properties of copper nanoparticles (CuNp)^21,35,42,61,66^ and bioactive glasses (BGNp)^31,67^ as single entities against *S. mutans*. In addition, there are studies that have shown antimicrobial activity against *S. mutans* of resin composites incorporating CuNp^68^ or BGNp.^31^ Recent studies have shown that copper incorporated bioglass matrix exhibits promising antimicrobial properties against both Gram-positive and Gram-negative bacteria.^2,45^ Although these elements have been incorporated individually into dental materials, and their combination has been successfully tested in scaffolds for bone regeneration.^33,62^ to the authors’ knowledge, there are no studies showing this capability when incorporated in combination into a resin composite system.

Thus, in the current study we can observe that the incorporation of BG/CuNp in a resin composite in concentrations of 5% and 10% increases the antimicrobial activity against *S. mutans* in comparison with the control (0%). Thus, the first null hypothesis was rejected. This result suggests that combined BG/CuNp could have synergistic antibacterial effects. However, the underlying synergistic antibacterial mechanisms of the multicomponent require further investigations.

Several mechanisms have been proposed by which the CuNp and BGNp could exert their antimicrobial effect.^13,44,48^ One theory is called ‘Trojan horse effect’, where the acidic lysosomal environment (pH 5.5) is capable of promoting nanoparticles degradation/corrosion, which converts core metals to ions and therefore toxic substances. Additionally, the impact of CuNp does not solely depend on the bacteria internalizing the nanoparticles; these nanoparticles can also locally alter the microenvironments surrounding the bacteria, resulting in the production of reactive oxygen species (ROS) or an increase in nanoparticle solubility.

This can lead to the dysfunction of other organelles and interactions with –SH groups of essential microbial enzymes, ultimately causing denaturation and inactivation of bacterial proteins and DNA damage, which disrupts DNA replication in the microorganisms.^13,22,50,69^ In this regard, recent studies from our research group have shown that incorporating CuNps into a polymeric system imparts antimicrobial properties after 1, 2, and 4 years of water storage, without diminishing the mechanical properties of the polymers.^20,21,41^

Meanwhile, the mechanisms that have been proposed to explain the antimicrobial action of BGNp refer to the increase in pH in an aqueous environment, creating an alkaline environment which inhibits bacterial growth, and the resulting shift in osmotic pressure caused by the release of ions such as sodium, calcium, phosphate, and silicate. These alterations can damage cellular structures and deactivate bacterial enzymes.^4,6^ Additionally, the increase in alkalinity may disrupt the proton motive force necessary for adenosine triphosphate (ATP) production.^24,32^ A recent study conducted by our research group demonstrated that incorporating 20% 45S5 bioactive glass into a polymeric material significantly increased the pH of the surrounding environment, in addition to promoting the deposition of hydroxyapatite and calcium carbonate (indicating bioactivity), and releasing substantial amounts of calcium ions.^6^

In this study, the viability of the GMSC cell line was tested. Several studies have shown the potential cytotoxicity of CuNp^27,63^ and BG,^52,65^ but little attention has been given to the effects of co-exposure, where nanoparticles of each material may also influence the processes or toxicity caused by the ions released by the other. In this sense, a recent study showed that the cytocompatibility of the experimental polymers doped with a copper-doped bioactive glass was significantly improved compared to the control, explained by the catalytic activity of released copper ions and to the lower monomer content.^28^ In the same line, this study shows that no differences were observed between the control resin composite (0%) and the BG/CuNp resin composites (5%, 10% and 20%), as well as between the experimental groups and the viability control, at day 0, 7 and 14. Thus, these results indicate that any potential cytotoxicity of the resin composites, both with and without BG/CuNp, even if minimal, is primarily due to the release of residual monomers rather than the concentration of BG/CuNp.

The present study demonstrated that the addition of copper-doped bioactive glass nanoparticles to resin composite systems at concentrations up to 10% could maintain (immediate time) or even improve (after 28 days) the ultimate tensile strength and microhardness, compared to the control. Thus, the second null hypothesis was rejected. Several studies have shown that microhardness increases when bioactive glass or copper particles are incorporated into polymeric systems,^6,20^ as there may be a positive correlation between the volume fraction of filler and the microhardness of resin-based materials, since filler particles are harder than the organic phase of the material. In the same context, recent studies have shown that the incorporation of bioactive glass or copper particles into polymeric systems^6,20^ does not negatively influence the ultimate tensile strength, which agrees with the results of this study. On the other hand, groups with BG/CuNp showed higher ultimate tensile strength and microhardness values than the control after 28 days. This could be explained by the catalytic effect of copper, which forms a tough and low-stress homogeneous glassy crosslinked network in the resin composite.^57^

For all BG/CuNp containing resin composite systems, similar values of resin–dentin bond strength were observed, along with a significant decrease in the nanoleakage values for both adhesive strategies. Several hypotheses may help to explain the nanoleakage results. Copper can increase the strength of the collagen network, one of the components of the hybrid layer, because the collagen cross-linking enzyme, lysyl oxidase (LOX), is copper-dependent,^36^ thus copper has an indirect effect as a cross-linking agent. This cross-linker action of copper nanoparticles alone may increase the resistance of collagen, making it less susceptible to the effects of proteolytic enzymes such as MMPs and CTs, thereby indirectly decreasing the immediate nanoleakage. In addition, bioactive glasses could reduce nanoleakage in the hybrid layer by replacing water with mineral crystals within unprotected collagen matrices,^1,53^ and by promoting the deposition of crystallite precipitates.^1,6^

As a final point, the 5% and 10% groups exhibit a degree of conversion comparable to that of the control group. This similarity can be attributed to the demonstrated compatibility of copper nanoparticles with the chemical components of methacrylate polymers and resin composite systems when incorporated.^49^ However, the 20% group shows a reduced degree of conversion compared to the control, regardless of the application mode. This is further supported by the lower microhardness observed in this group in comparison to the control. It is possible to hypothesize that this effect may be due to the high concentration of BG/CuNp, which could function as a plasticizer within the polymer network.

One of the limitations of this study is that, as it was conducted in a laboratory setting, the results obtained cannot be extrapolated to clinical practice. In addition, a long-term follow-up of these restorations is necessary to determine whether the improvement in the adhesive properties and stability of the hybrid layer is maintained over time.

Furthermore, the measurement of antimicrobial activity, considering only one type of bacteria, partially supports the proposal of a resin composite to prevent secondary caries. However, a more comprehensive approach, taking into account the oral microbiome, would provide a better understanding of the phenomenon. Moreover, since it is estimated that 40–50% of bacteria present in dental biofilms are not cultivable, more sophisticated methods, such as DNA sequencing-based techniques, should be employed to gain a deeper understanding of the effects of restorative materials on dental biofilm and vice versa.^40^

## CONCLUSION

The present study demonstrated that the addition of copper-doped bioactive glass nanoparticles to resin composite systems in concentrations up to 10 wt% is a feasible approach to enhancing antimicrobial properties and improving the stability of the resin–dentin interface, without causing significant biological hazards.

### Clinical Relevance

This is the first study to demonstrate that the addition of copper-doped bioactive glass nanoparticles in concentrations up to 10 wt% into a resin composite is a feasible approach and may serve as an alternative for adhesive interfaces with antimicrobial properties and fewer defects in the resin–dentin interface.

### Conflict of Interest

The authors declare no competing financial interest.

### Acknowledgment

This study was conducted by Romina Aliaga-Galvez as part of her PhD degree at the Universidad de los Andes (UAndes), Santiago, Chile. This project was funded by ANID (Agencia Nacional de Investigación y Desarrollo) through Initiation Fondecyt (Fondo Nacional de Desarrollo Científico y Tecnológico – Chile) grant number 11221070 (Chile; MFG). Additionally, this study received partial support from the National Council for Scientific and Technological Development (CNPq) under grant 308286/2019-7 (Brazil; ADL) and from the Coordenação de Aperfeiçoamento de Pessoal de Nível Superior – Brasil (CAPES) under Finance Code 001.
